# Research on multi-model imaging machine learning for distinguishing early hepatocellular carcinoma

**DOI:** 10.1186/s12885-024-12109-9

**Published:** 2024-03-21

**Authors:** Ya Ma, Yue Gong, QingTao Qiu, Changsheng Ma, Shuang Yu

**Affiliations:** 1 Department of Graduate, Shandong First Medical University, Shandong Academy of Medical Sciences, Jinan, China; 2 Department of Radiation Physics, Department of Radiotherapy, Shandong Cancer Hospital and Institute, Shandong First Medical University, Shandong Academy of Medical Sciences, Shandong Province, 250117 Jinan, China; 3 Department of Hematology, Qilu Hospital of Shandong University, 250012 Jinan, China

**Keywords:** Radiomics, Hepatocellular Carcinoma, Computed tomography, Magnetic resonance imaging

## Abstract

**Objective:**

To investigate the value of differential diagnosis of hepatocellular carcinoma (HCC) and non-hepatocellular carcinoma (non-HCC) based on CT and MR multiphase radiomics combined with different machine learning models and compare the diagnostic efficacy between different radiomics models.

**Background:**

Primary liver cancer is one of the most common clinical malignancies, hepatocellular carcinoma (HCC) is the most common subtype of primary liver cancer, accounting for approximately 90% of cases. A clear diagnosis of HCC is important for the individualized treatment of patients with HCC. However, more sophisticated diagnostic modalities need to be explored.

**Methods:**

This retrospective study included 211 patients with liver lesions: 97 HCC and 124 non-hepatocellular carcinoma (non-HCC) who underwent CT and MRI. Imaging data were used to obtain imaging features of lesions and radiomics regions of interest (ROI). The extracted imaging features were combined to construct different radiomics models. The clinical data and imaging features were then combined with radiomics features to construct the combined models. Support Vector Machine (SVM), K-nearest Neighbor (KNN), RandomForest (RF), eXtreme Gradient Boosting (XGBoost), Light Gradient Boosting Machine (LightGBM), Multilayer Perceptron (MLP) six machine learning models were used for training. Five-fold cross-validation was used to train the models, and ROC curves were used to analyze the diagnostic efficacy of each model and calculate the accuracy rate. Model training and efficacy test were performed as before.

**Results:**

Statistical analysis showed that some clinical data (gender and concomitant cirrhosis) and imaging features (presence of envelope, marked enhancement in the arterial phase, rapid contouring in the portal phase, uniform density/signal and concomitant steatosis) were statistical differences (*P* < 0.001). The results of machine learning models showed that KNN had the best diagnostic efficacy. The results of the combined model showed that SVM had the best diagnostic efficacy, indicating that the combined model (accuracy 0.824) had better diagnostic efficacy than the radiomics-only model.

**Conclusions:**

Our results demonstrate that the radiomic features of CT and MRI combined with machine learning models enable differential diagnosis of HCC and non-HCC (malignant, benign). The diagnostic model with dual radiomic had better diagnostic efficacy. The combined model was superior to the radiomic model alone.

**Supplementary Information:**

The online version contains supplementary material available at 10.1186/s12885-024-12109-9.

## Introduction

Primary liver cancer is one of the most common clinical malignancies, with the sixth incidence rate and the third mortality rate among all cancers [1]. HCC is the most common subtype of primary liver cancer, accounting for approximately 90% of cases [2]. The atypical clinical symptoms of HCC in the early stage and the rapid development in the middle and late stages are the main reasons for the high mortality rate of HCC. Therefore, a clear diagnosis of HCC is important for the individualized treatment of patients with HCC [3].

Many researchers have found that imaging features of HCC are of great research value for differential diagnosis. Contrast-enhanced ultrasound (CEUS) was reported to aid in the differential diagnosis of malignant and benign liver cancer [4]. 64-slice spiral CT was reported to provides more adequate imaging evidence for the clinical diagnosis of HCC and hepatic focal hyperplastic nodules than conventional ultrasound, it can effectively identify benign and malignant tumors, and have a high sensitivity to the diagnosis of small lesions [5]. However, both CEUS and enhanced CT can only diagnose HCC anatomically. As research continues, many researchers are now suggesting that functional magnetic resonance imaging has great potential to help in differential diagnosis. For example, several studies of HCC based on ultrasound, CT and MR images found that MR images of the hepatobiliary stage were the most sensitive [6]. However, these studies still do not provide a definitive diagnosis of HCC.

Radiomics is a technique for converting medical images into mineable data, based on a method for quantitative features. Thanks to advances in medical image acquisition and analysis, it is now possible to objectively and quantitatively describe the phenotype of the tumor [7]. However, several challenges must be addressed before radiological features can be applied in clinical practice, including the standardization and stability of the selected features [8]. One of the main challenges in radiomics is the reproducibility of quantitative imaging features [9]. To stable and unbiased descriptions, it is essential to quantify various imaging features objectively and reproducibly. The potential redundancy of image features is another major challenge in radiomics [10]. The redundant features can add complexity to radiological studies [11]. Thus, the purpose of our study was to evaluate the value of preoperative CT and MR multi-stage radiomics in the clinical diagnosis of HCC, and further compare the diagnostic efficacy of between different radiomics models of CT, MR and CT + MR.

## Materials and methods

### Patients

The protocol for this study was approved by the Institutional Review Committee of the Shandong First Medical University Affiliated Cancer Hospital Ethics Committee. The ethics filing number is SDTHEC2020010008. The patients were assigned to the HCC group according to the “Standard for diagnosis and treatment of primary liver cancer (2022 edition)”, the other patients with liver lesions (include hepatic hamangioma, hepatic adenoma, liver metastases and intrahepatic cholangiocarcinoma) were assigned to the non-HCC group (malignant and benign group). The imaging images of 211 patients in the Tumor Hospital of Shandong Province (main center) between February 2017 and November 2020 and the Second Affiliated Hospital of Shandong First Medical University (sub center) between July 2021 and June 2022 were analyzed. Inclusion criteria: (1) meeting the clinical diagnostic criteria for liver lesions; (2) doing the abdominal CT and MR plain scanning and contrast-enhanced imaging prior to treatment. Exclusion criteria: (1) CT and MR examinations time exceeding 1 month; (2) incomplete imaging data; (3) poor image quality; (4) multiple lesions. Clinical data were collected, including age, gender and history of cirrhosis.

### Image acquisition

Plain CT scanning and contrast-enhanced scanning utilized multi-layer spiral CT (Aquilion 16, Toshiba Medical Systems Corporation) and 256-row CT (Brilliance iCT, Philips Medical Systems). The scanning parameters were as follow: tube voltage 125kVp; tube current 320mAs; gap 0.95 mm; thickness 2-5 mm; reconstruction gap 2 mm. Iohexol was injected through the upper limb vein at a dose of 1.5 ml/kg and at a rate of 3.0 ml/s. Dynamic contrast-enhanced scans were performed at 25-30s, 60-65s and 120-140s after contrast injection.

Plain MR scanning and contrast-enhanced scanning utilized 3.0TMR US GE Discovery MR750W and MR750 scanners, an 8-channel body phased array coil, plus breathing hose, the scanning sequence included the cross-sectional T2WI fat suppression sequence: repetition time (TR) 10000ms, echo time (TE) 85ms, thickness 5 mm, gap 2 mm, FOV 38 cm×38 cm, matrix 320 × 320. Breath-holding transverse axis T1WI rapid volume acquisition plain scan and enhancement sequence: TR 4.1ms, TE 1.9ms, thickness 5 mm, gap 2.5 mm, FOV 40 cm×32 cm, matrix 320 × 244. Magnevist Solution was injected through the cubital vein at a dose of 0.1mmol/kg and at a rate of 2.0 ml/s, and then 20 ml normal saline was injected into tube at the same rate. Dynamic three-stage enhanced scanning of the entire liver was performed in 20-25s, 50-65s and 160-180s after injecting the contrast medium, each scanning required a breath-hold for 13s. Axial diffusion-weighted imaging (DWI; b = 50, 800s/mm^2^).

### Image analysis

After transferring all the patients’ image data to the PACS system, the evaluation of all the patients’ image information was done independently by two abdominal diagnosticians (with 3 and 10 years of diagnostic experience), blinded to the patients’ clinical information. After observing all images of the patient, two physicians evaluated the routine imaging features of the lesion.

### Tumor segmentation

**Fig. 1:**
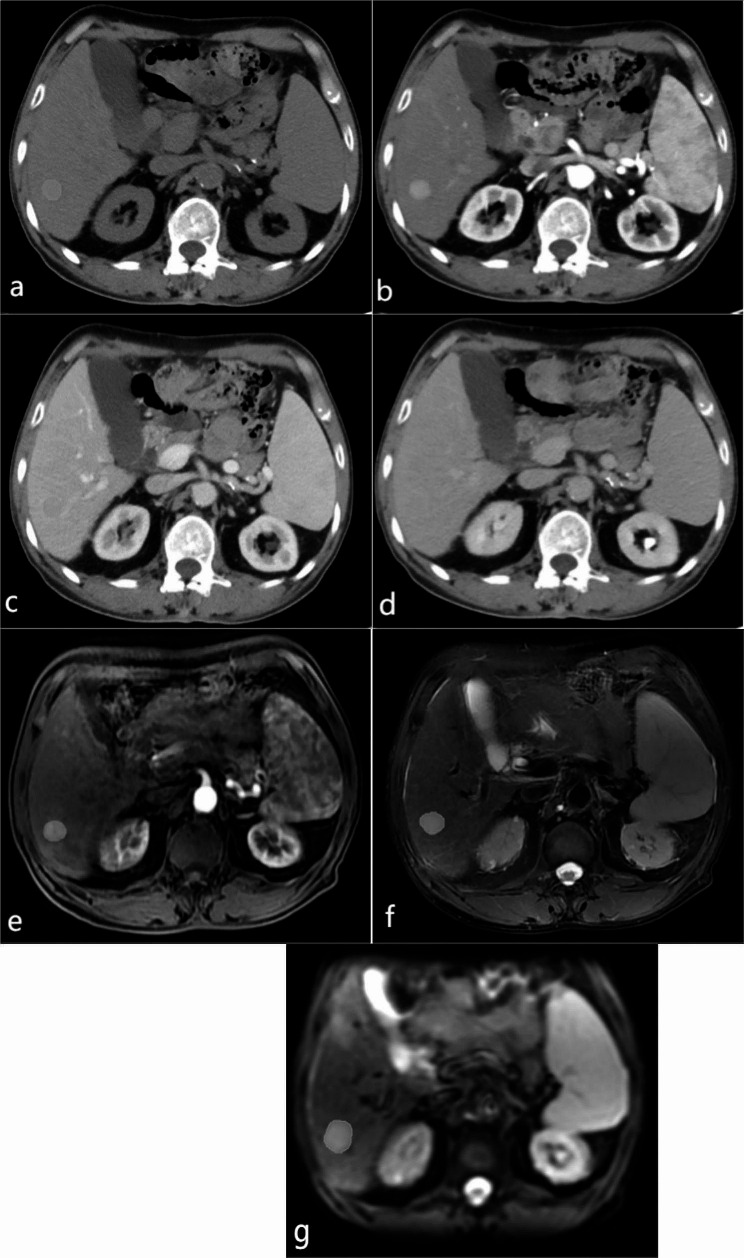
A 62-year-old male patient with HCC. A-D show CT axial images: **A** plain scanning phase, **B** arterial phase, **C** portal venous phase, **D** delayed phase; **E-G** show MRI axial images: **E** arterial phase, **F** T2WI, **G** DWI

The original images of all CT and MRI were imported separately into 3Dslicer software to outline the ROI by an abdominal imaging radiologist with 3 years of experience, blinded to the patient’s pathology or any clinical information. Volume of interest (VOI) including complete information of the lesion was obtained. The ROI was then calibrated by other abdominal imaging radiologist with over 10 years of experience to finalize the segmentation results. Another imaging physician randomly selected 30 patients for a second VOI manual segmentation and extraction to assess consistency between observers. (Fig. 1) The intraclass correlation coefficient (ICC) between 0.75 and 1 indicates good agreement.

### Feature extraction and dimension reduction

Radiomics features were extracted using open-source library PyRadiomics. A total of 749 features were extracted. Before these features were filtered, normalization of data using method of maximum-minimum divided the scale orders of these features as 0–1. Feature reduction was performed by the Spearman correlation and least absolute shrinkage and selection operator (LASSO) to obtain the most useful diagnostic features. By integrating the filtering features separately, the combined features of CT, MR and CT + MR were ultimately obtained.

### Model construction and evaluation

Machine learning models (CT radiomics models, MR radiomics models, and CT + MR dual radiomics models) were constructed using the above filtering features. Then the three combinations were combined with the patient’s clinical data to construct models of radiomics and clinic (CT-CF, MR-CF and CT + MR-CF). This given a total of 6 models. Five-fold cross-validation was applied in training group and the average of the five results was obtained. The receiver operating characteristic curve (ROC) was used to predictive performance of each model and the area under the ROC curve (AUC) and accuracy were calculated. The diagnostic efficacy of the models in the training group was evaluated. The sub-center patients were then used as the test group to test the diagnostic efficacy of the model in the test group.

### Statistical analysis

The SPSS 26.0 software was used for statistical analysis of clinical data and imaging features. The Shapiro-Wilk test was used to test whether the measurement data conformed to a normal distribution, and data that followed a normal distribution overall were represented by means (standard deviation), an F-test was used. Others were expressed as medians (interquartile spacing), Multisample rank sum test was used. The χ2 test or Fisher’s test was used for the counting data. *P* < 0.05 means that the difference is statistically significant.

## Results

### Clinical characteristics of patients

**Fig. 2:**
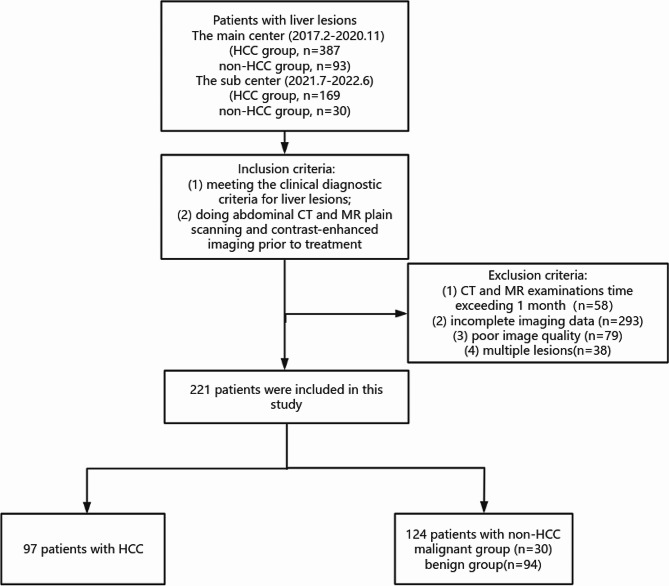
Flowchart shows the patient selection process

In this study, 221 patients who met the inclusion criteria were divided into HCC and non-HCC groups (benign and malignant groups). Fig. 2 presents the flow diagram of patient selection process. The analytical results of the measurement data showed a significant statistical difference in age in the HCC group and non-HCC group (*p* = 0.041) and a significant statistical difference in the maximum diameter of the tumor in the malignant and benign groups of non-HCC (*p* = 0.003), but there were no statistical difference in HCC group and malignant and benign groups in the non-HCC group (*p* = 1.000 and *p* = 0.136, *p* = 0.072 and *p* = 0.064). The analytical results of the classified data showed a significant statistical difference in the gender, history of cirrhosis and in the imaging features such as presence of capsule, significant enhancement in the arterial phase, contouring, homogeneous density/signal of the lesion, and steatosis in the three groups.(Table 1).

**Table 1:** Clinical characteristics of the patients in the training and test groups

Features	The group of HCC(*n* = 97)	The group of non-HCC (*n* = 124)		The test value (*H*-value)	*P*-value
		Malignant group (*n* = 30)	Benign group (*n* = 94)		
Age(year)Mean(SD)	57.64(9.1)	53.17(11.5)	58.83(11.7)	3.237	0.041
The maximum diameter of the tumor (mm)M(IQR)	35.0(23.5,58.5)	40.5(25.0,66.25)	25.5(15.75,50.25)	11.6	0.003
Gender(%)				16.702	< 0.001
Male	78(80.4)	13(43.3)	58(61.7)		
Female	19(19.6)	17(56.7)	36(38.3)		
Cirrhosis(%)				73.794	<0.001
Yes	79(81.4)	5(16.7)	24(25.5)		
No	18(18.6)	25(83.3)	70(74.5)		
Place (%)				2.967	0.227
Right lobe	85(87.6)	23(76.7)	75(79.8)		
Left lobe	12(12.4)	7(23.3)	19(20.2)		
Capsule(%)				66.913	<0.001
Yes	80(82.4)	8(26.7)	25(26.6)		
No	17(17.6)	22(73.3)	69(73.4)		
Significant enhancement of the arterial phase (%)				39.442	<0.001
Yes	95(97.9)	20(66.7)	58(61.7)		
No	2(2.1)	10(33.3)	36(38.3)		
Contouring(%)				84.099	<0.001
Yes	86(88.6)	7(23.3)	26(27.7)		
No	11(11.4)	23(76.7)	68(72.3)		
Uniform (%)				62.951	<0.001
Yes	5(5.1)	10(33.3)	58(61.7)		
No	92(94.9)	20(66.7)	36(38.3)		
Steatosis(%)				42.876	<0.001
Yes	72(74.2)	4(13.3)	37(39.4)		
No	25(25.8)	26(86.7)	57(60.6)		

### Clinical model establishment and evaluation

Independent risk factors were obtained by multivariate logistics regression analysis of statistically significant clinical data and imaging characteristics (*P* < 0.001 in Table 1). Based on the factors, a clinical diagnostic model was established and the model was performed by five-fold cross-validation. The prediction results of the differential diagnosis model for HCC in the training and test groups are shown in the Table 2.

**Table 2:** Clinical features combined with machine learning models for diagnostic performance

	Accuracy	
	Training group	Test group
SVM	0.701	0.750
KNN	0.740	0.773
RandomForest	0.870	0.727
XGBoost	0.842	0.795
LightGBM	0.701	0.750
MLP	0.689	0.727

### Radiomics feature screening results

In the VOI obtained from the preoperative segmentation of the CT and MRI images in 221 patients, 107 features were extracted in each of the 7 phases. After screening with ICC > 0.75, the image feature parameters of each period were fused, comp1 = CT, comp2 = MR, comp3 = CT + MR. The three groups of parameters were then screened for Spearman correlation coefficient to retain characteristic parameters with corr > 0.9. The training group was done five-fold cross-validation, parameters after dimension reduction by the lasso model were filtered and the coefficients with coefficients>0 were used to construct machine learning models.

### Comparison of diagnostic efficacy between different radiomics models

**Fig. 3:**
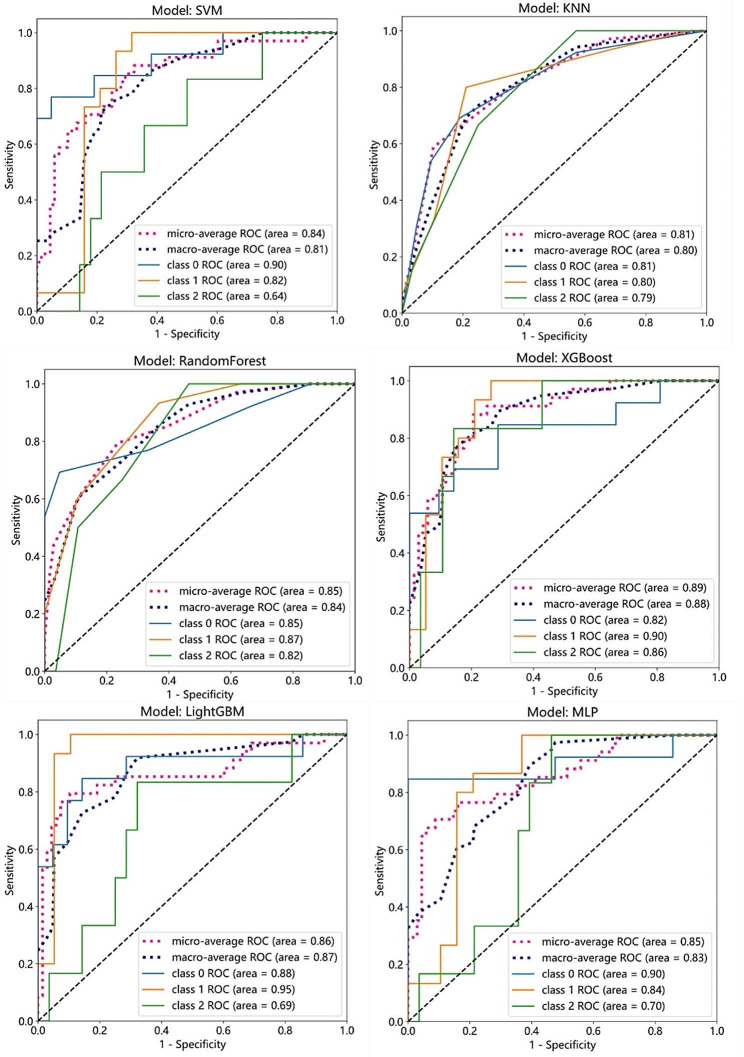
Area under the ROC curve of a diagnostic model. Area under the ROC curve for constructing machine leaning model diagnostic models based on CT + MR dual radiomics features in the training and test groups. **Class 0** Benign group. **Class 1** HCC group. **Class 2** Malignant group

The main center serves as the training group and the sub center as the test group. The screened radiomics features from each group were combined to construct multiple machine models of the training group CT, MR and CT + MR. The machine-learning methods include the SVM, KNN, RandomForest, XGBoost, LightGBM and MPL. (Table 3) The results showed that many machine learning models had good diagnostic efficiency. In the test group, the diagnostic efficacy of CT + MR dual radiomics model was higher than the other two groups. The efficacy of the CT + MR dual radiomics models in differentiating HCC and non-HCC (malignant and benign groups) is shown in the Fig. 3.

**Table 3:** Diagnostic efficacy of pure radiomics combined with different machine learning models

		Accuracy	
Classifier		Training group	Test group
SVM			
	CT	0.707	0.641
	MR	0.753	0.676
	CT + MR	0.752	0.711
KNN			
	CT	0.650	0.692
	MR	0.662	0.647
	CT + MR	0.753	0.737
RandomForest			
	CT	0.994	0.615
	MR	0.967	0.579
	CT + MR	0.985	0.706
XGBoost			
	CT	1.000	0.615
	MR	1.000	0.658
	CT + MR	1.000	0.706
LightGBM			
	CT	0.790	0.590
	MR	0.787	0.553
	CT + MR	0.774	0.794
MLP			
	CT	0.662	0.615
	MR	0.692	0.676
	CT + MR	0.760	0.710

**Fig. 4:**
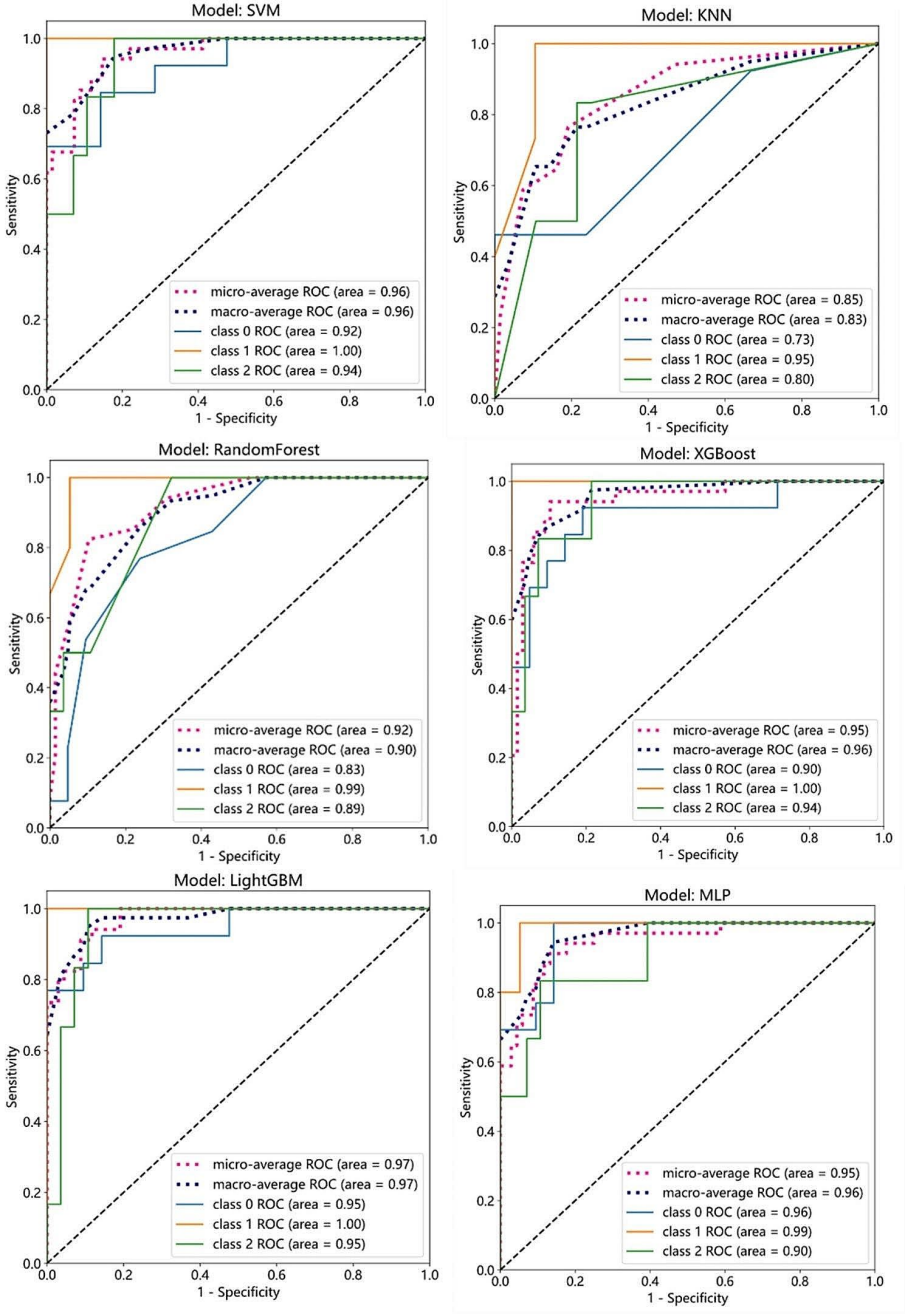
Area under the ROC curve of a diagnostic model. Area under the ROC curve of a diagnostic model based on CT + MR dual radiomics features combined with clinical information and imaging features to construct a machine learning model in the training group and test group. **Class 0** Benign group. **Class 1** HCC group. **Class 2** Malignant group

The three groups of imaging radiomics characteristics selected after the above screening were combined with clinical data and imaging features to construct the clinical-radiomics model. (Table 4) The results showed that the clinical-radiomics model had better diagnostic efficacy than the radiomics model alone. The efficacy of the clinical-radiomics model based on CT + MR dual radiomics features in differentiating HCC and non-HCC (malignant and benign groups) was shown in the Fig. 4.

**Table 4:** Diagnostic efficacy of joint features combined with machine learning models

		Accuracy	
Classifier		Training group	Test group
SVM			
	CT	0.834	0.744
	MR	0.887	0.789
	CT + MR	0.887	0.824
KNN			
	CT	0.721	0.697
	MR	0.727	0.647
	CT + MR	0.774	0.711
RandomForest			
	CT	0.981	0.743
	MR	0.980	0.605
	CT + MR	0.985	0.765
XGBoost			
	CT	1.000	0.744
	MR	1.000	0.711
	CT + MR	1.000	0.882
LightGBM			
	CT	0.815	0.744
	MR	0.833	0.737
	CT + MR	0.835	0.824
MLP			
	CT	0.771	0.718
	MR	0.800	0.658
	CT + MR	0.782	0.824

## Discussion

Three mainly pathological types of primary liver cancer (HCC, intrahepatic cholangiellular carcinoma, and mixed HCC-cholangiocarcinoma) are different in various aspects, such as nosogenesis, biological behaviour, histopathology, treatment methods and prognosis. Pathological diagnosis has always been regarded as the gold standard for neoplastic lesions, but the anatomical peculiarities of the liver make it difficult to sample [12]. Most tumors require confirmation of examination findings by tissue sampling before treatment, but HCC can be diagnosed through non-invasive examination [13].

In this study, the imaging data and clinical data of 221 patients were analyzed. Some scholars found that the maximum diameter of the tumor was an independent risk factor for identifying benign and malignant tumors of the liver [14]. In this study, the difference in maximum tumour diameter was statistically significant in the benign and malignant groups of non-HCC, but not in HCC and non-HCC. Gender was statistically significant in HCC and non-HCC groups, but age was no statistically significant in the HCC and the other two groups, which may be related to the inclusion of metastases and cholangiocarcinoma cases in the non-HCC group in our study. Studies shown that HCC, liver metastases and intrahepatic cholangiocarcinomas tended to occur in medium-elderly people [14, 15]. Some imaging features have differential diagnostic value, such as capsule, heterogeneity, arterial phase of significant enhancement, contouring, and the presence of steatosis. Strengthening of the capsule may represent a pseudo-capsule. The authenticity of the capsule cannot be distinguished by imaging and can only be assessed by pathology [16–19]. One study shown that the sensitivity and specificity of MR for capsules diagnosis were 94.0% and 73.2%, respectively [16]. Capsules formation is an important pathological feature in the progression of HCC, so, the presence of capsules had clinically significant for the differentiation of HCC from cholangiocarcinoma, which shared the results of this study. Arterial phase of significant enhancement is an important biological feature of HCC. Six studies reported that arterial phase of significant enhancement was more sensitive than other dynamic contrast-enhancing features for the diagnosis of progressive (i.e., malignant tumours capable of invading blood vessels and metastases) HCC, with reported the sensitivity ranging from 65–96% [20, 21]. Therefore, this study included the manifestation of arterial phase of significant enhancement when evaluating the imaging features of the lesions, and the results showed a statistically significant difference between the two groups. However, the specificity of the arterial phase of significant enhancement for the diagnosis of HCC was low, and some scholars suggested that combining it with contouring to improve the detection rate of HCC [22]. For most imaging algorithms, ‘contouring’ is considered a strong predictor and the main criterion for HCC [23–27]. This study found that the contouring was statistically significant in HCC and non-HCC. This study also included the homogeneity of the signal/density of the lesions and the presence of steatosis. The results suggested that the presence of steatosis had differential diagnostic significance, some studies [28] pointed out that steatosis in HCC was usually associated with ischemia, some scholars suggested that the presence of fat in the lesion should be included in the LI-RADS auxiliary diagnostic criteria.

This study showed that the CT + MR dual radiomics model was slightly better than the single radiomics models in the test group. Radiomics can effectively improve the correlation between images and pathology and clinic by mining the feature information of the high dimension in the image. Some scholars [29] found that the model based on textured features of patient CT scan could effectively distinguish between hemangioma and HCC. A recent study found that a model based on the arterial phase and the portal venous phase can distinguish between necrotic liver cancer and septic liver abscess [30]. However, the above studies did not combine plain and enhanced CT images, so imagiomics characteristic information of some lesions may be missed. In this study, it was concluded that CT scans combined with triple enhancement images had good diagnostic efficiency in distinguishing liver cancers, but the diagnostic efficiency of this model was reduced in the test group, probably because the test group was derived from the sub-center and the distribution of diseases in the non-HCC group was not equal. However, in the test group, similar to the results of other researches, the combined clinical model of the radiomics was better than the simple radiomics model.

Magnetic resonance imaging (MRI) is important in the early definitive diagnosis of diseases such as HCC. In recent years, many researchers found the importance of MRI radiomics models in differentiating lesions such as HCC and intrahepatic cholangiocarcinoma. Feng Huang et al. [31] constructed the differential diagnosis model of HCC and intrahepatic cholangiocyte carcinoma based on T 2 WI images, the results showed that the AUC of the model in the training group and test group was 0.90 and 0.91, respectively, and the DCA curve Nomo model had clinical application value. Zongren Ding et al. [32], based on the MR images of Gd-DTPA, carried out the differential diagnosis of HCC and focal nodule hyperplasia in the background of non-cirrhosis, and constructed the combined clinical model and clinical-radiomics model, respectively. This can be obtained that the diagnostic efficacy of the combined model in the test group was better than that of the clinical model was concluded that the combined clinical-radiomics model provided a non-invasive and quantitative method to differentiate HCC from focal nodular hyperplasia. The results of most studies were also similar to our results, which showed that the combined model outperformed the radiomics-only model.

We must acknowledge some limitations. Firstly, as a retrospective study, there may have been selection bias in enrolling patients, and our study includes a relatively small sample size. In addition, only some of clinical data and imaging features of patients were evaluated in this study, and no laboratory indicators were included. Then, this study did not explore the pathological aspects of the lesions. Finally, we manually outlined the entire area of the lesion by the imaging physician, this work was very time-consuming. By future further studies, the above mentioned limitations could be overcome by increasing the sample size, doing prospective research, obtaining pathological indicators, establishing a more comprehensive diagnostic model by combining more indicators, using the AI and so on.

## Conclusions

In summary, our findings demonstrate that according to the CT and MR image analysis of patients with liver lesions, the differential diagnosis of HCC and non-HCC (malignant group and benign group) can be made and the feasibility of CT + MR dual radiomics model for preoperative diagnosis of HCC was initially proved. On the basis of our results, we recommend that combined with these characteristics could better inform current clinical practice for the diagnosis and treatment of HCC, and favor further research of the AI recognition classification model of liver lesions. Further research is required to strengthen this evidence.

### Electronic supplementary material

Below is the link to the electronic supplementary material.

Supplementary Material 1

Supplementary Material 2

## Data Availability

The datasets used and analyzed during the current study available from the corresponding author on reasonable request (machangsheng_2000@126.com).

## Abbreviations

CT: Computed Tomography
MRI: Magnetic Resonance Imaging
DWI: Diffusion Weighted Imaging
T2WI: T2-Weighted Imaging
ROI: Region of Interest
VOI: Volume of Interest
ROC: Receiver Operating Characteristic
AUC: Area Under the Curve
ICC: Intraclass Correlation Coefficient
SVM: Support Vector Machine
KNN: K-Nearest Neighbor
RF: Random Forest
XGBoost: Extreme Gradient Boosting
LightGBM: Light Gradient Boosting Machine
MLP: Multilayer Perceptron
GLRLM: Gray Level Run Length Matrix
GLCM: Gray Level Co-occurrence Matrix
GLSZM: Gray Level Size Zone Matrix
NGTDM: Neighborhood Gray-Tone Difference Matrix
GLDM: Gray Level Dependence Matrix
CEUS: Contrast-enhanced Ultrasound
TR: Repetition Time
TE: Echo Time
