# Supplementary material for: Research on multi-model imaging machine learning for distinguishing early hepatocellular carcinoma
Source: BMC Cancer. 2024 Mar 21;24:363. doi: 10.1186/s12885-024-12109-9 (PMC10956394; doi:10.1186/s12885-024-12109-9)
Supplement: Supplementary file 1 — Supplementary Material 1 [file 12885_2024_12109_MOESM1_ESM.docx]

**
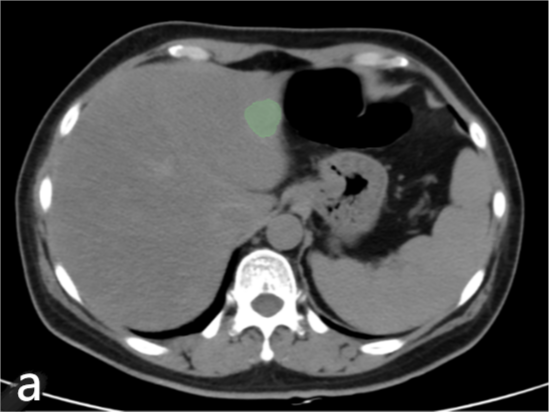

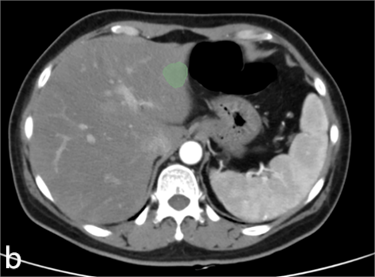
**

**
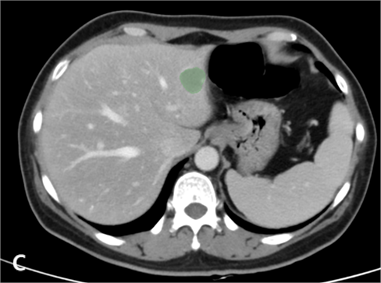

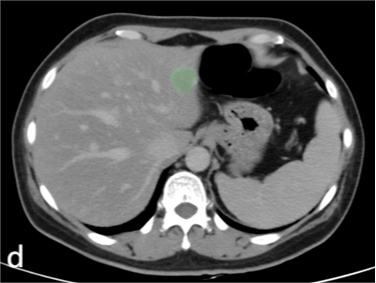
**

**
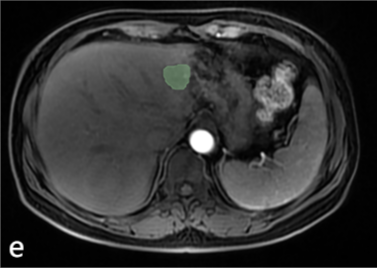

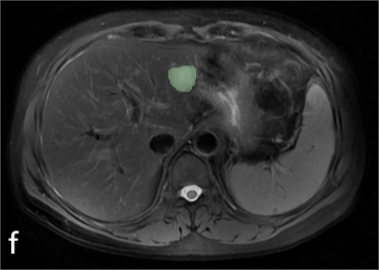
**

**
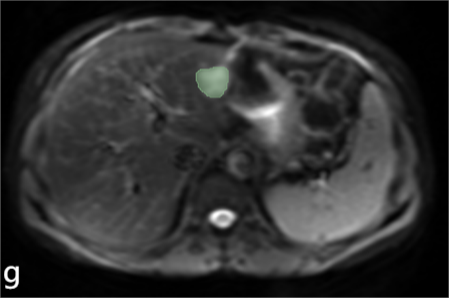
**

**Fig.S1 52-year-old female patient with hepatic hemangioma**

**a-d** show CT axial images: **a** plain scanning phase, **b** arterial phase, **c** portal venous phase, **d** delayed phase; **e-g** show MRI axial images: **e** arterial phase, **f** T2WI, **g** DWI.

**
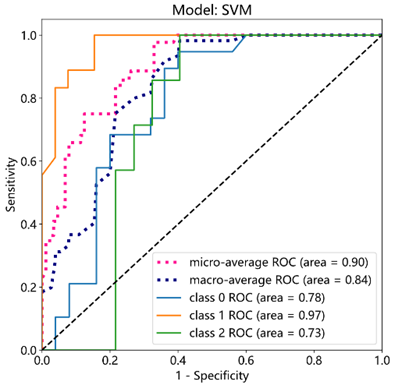

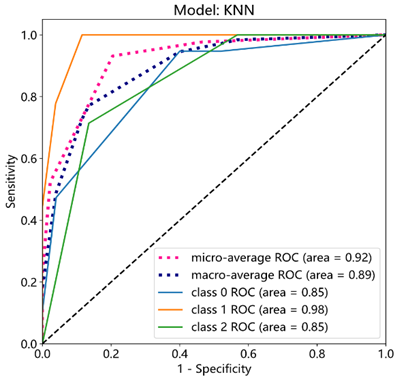
**

**
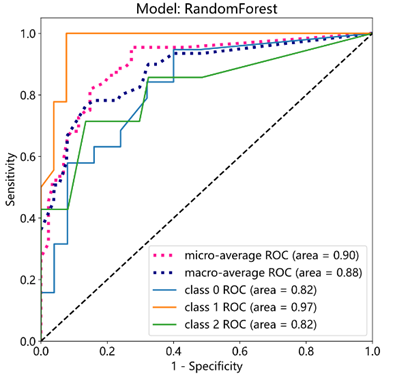

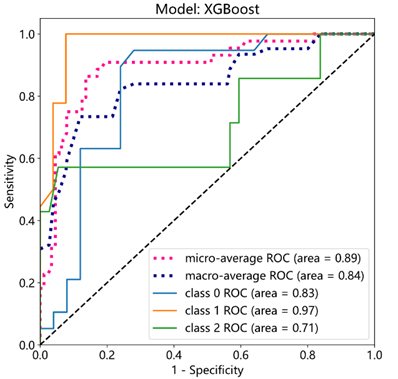
**

**
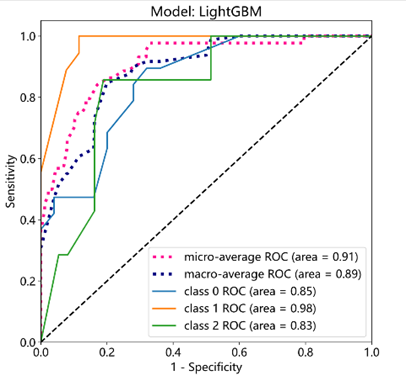

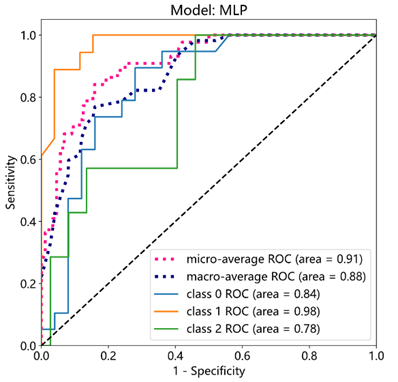
**

**Fig.S2** **Area under the ROC curve of a diagnostic model.** Area under the ROC curve of the training and test groups based on the screened clinical and different simultaneous phase imaging feature parameters to construct machine learning models **Class 0** Benign group. **Class 1** HCC group. **Class 2** Malignant group

**
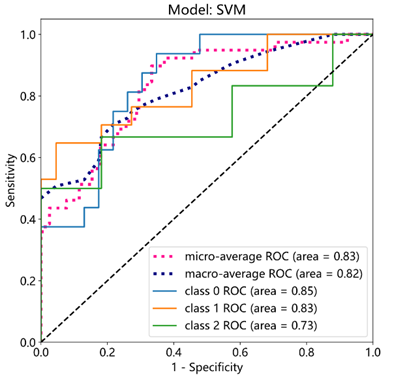

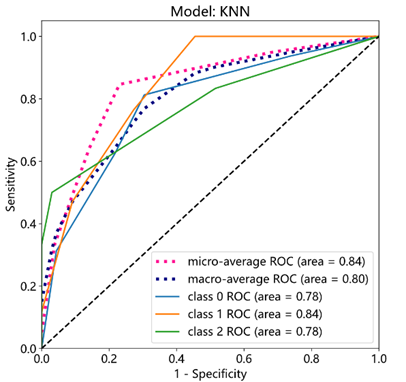
**

**
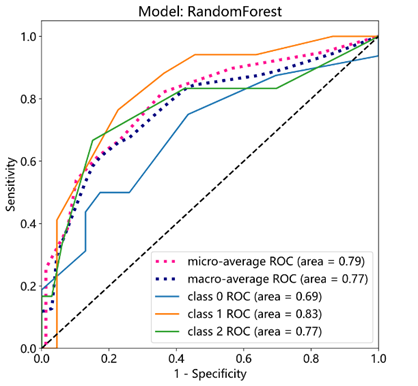

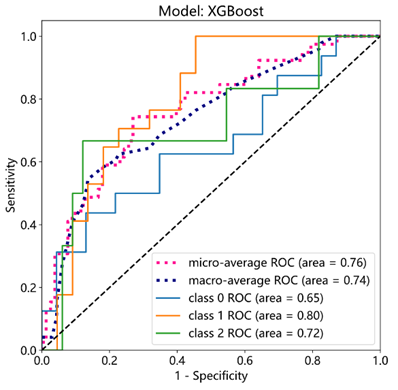
**

**
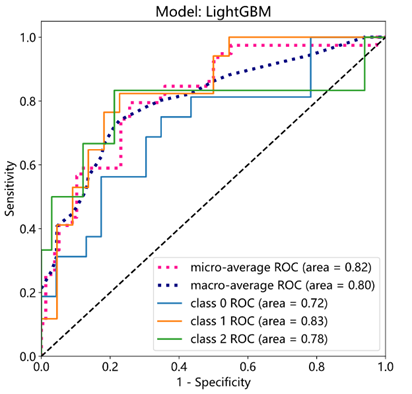

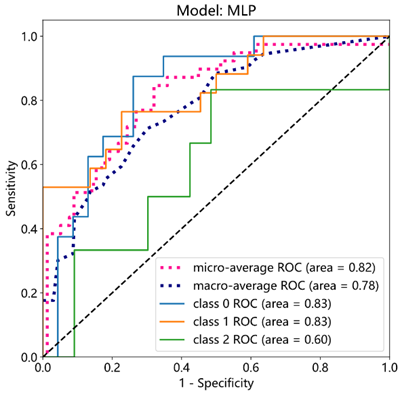
**

**Fig.S3 Area under the ROC curve of a diagnostic model.** Area under the ROC curve for constructing machine learning model diagnostic models based on CT radiomics features in the training and test groups **Class 0** Benign group. **Class 1** HCC group. **Class 2** Malignant group

**
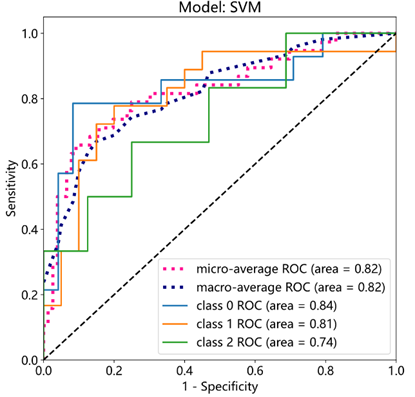

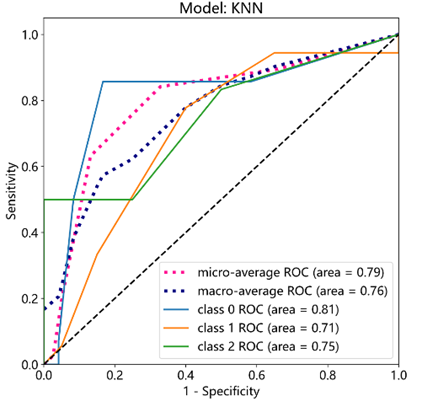
**

**
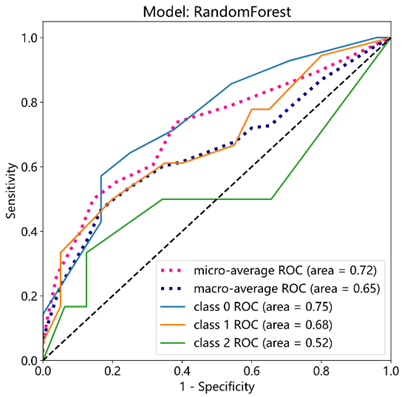

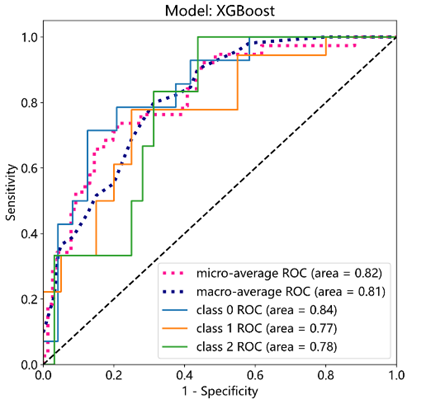
**

**
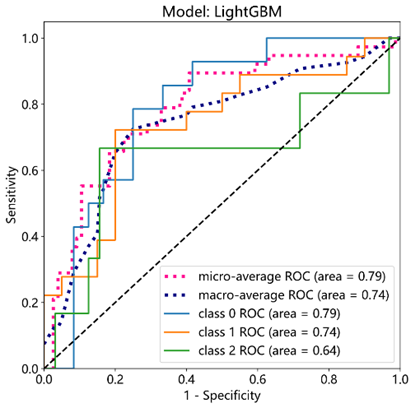

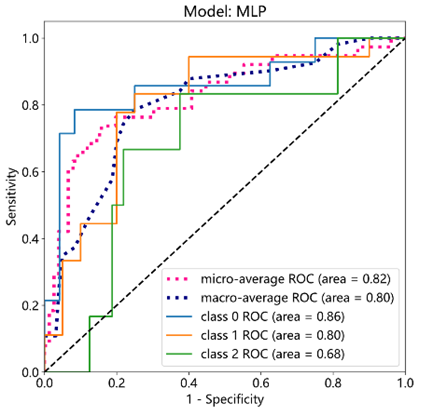
**

**Fig.S4 Area under the ROC curve of a diagnostic model.** Area under the ROC curve for constructing machine learning model diagnostic models based on MR radiomics features in the training and test groups **Class 0** Benign group. **Class 1** HCC group. **Class 2** Malignant group

**
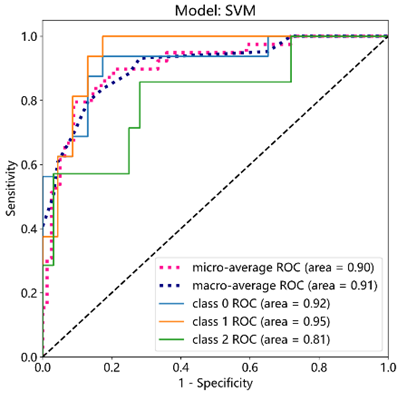

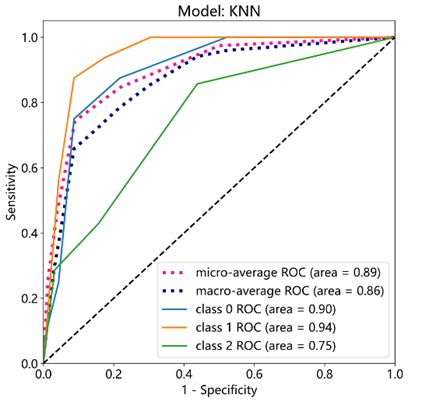
**

**
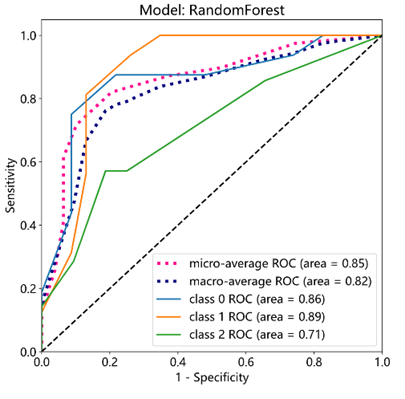

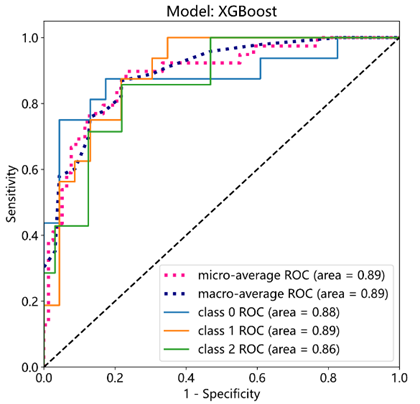
**

**
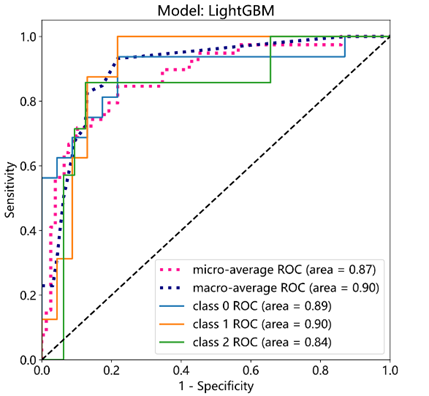

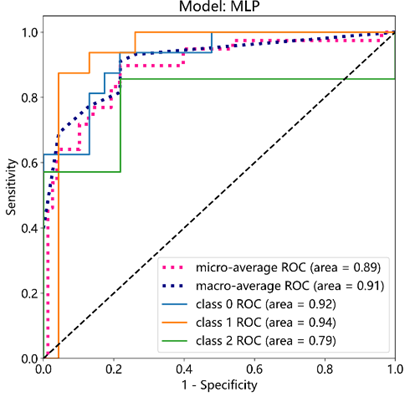
**

**Fig.S5 Area under the ROC curve of a diagnostic model.** Area under the ROC curve of a diagnostic model based on CT radiomics features combined with clinical information and imaging features to construct a machine learning model in the training and test groups **Class 0** Benign group. **Class 1** HCC group. **Class 2** Malignant group

**
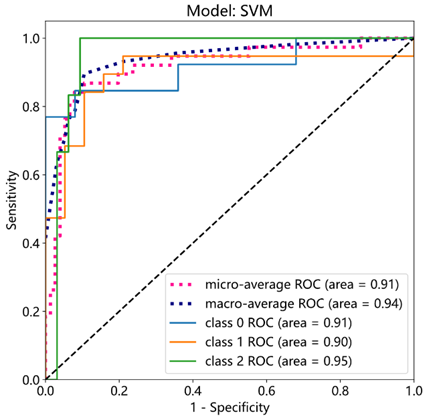

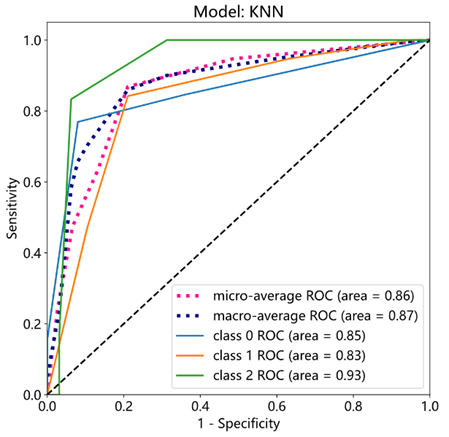
**

**
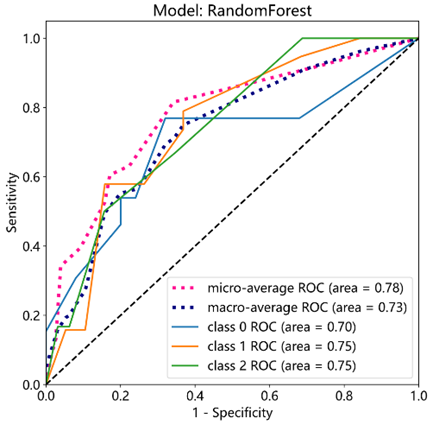

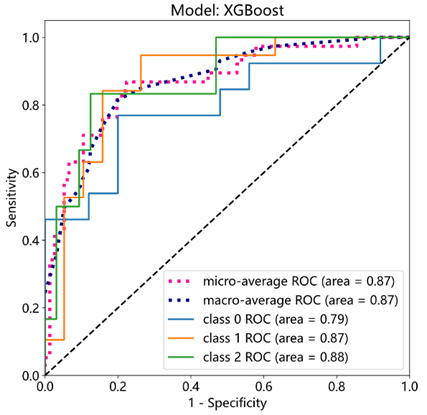
**

**
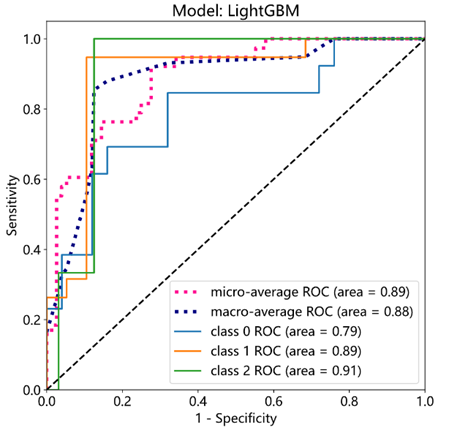

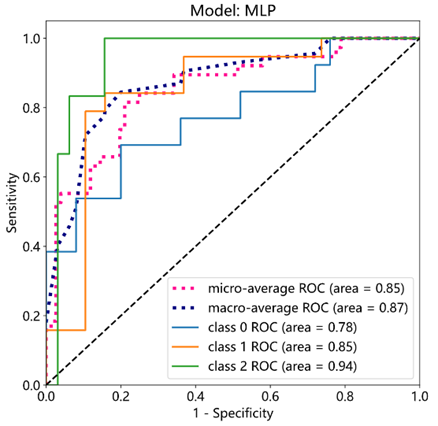
**

**Fig.S6 Area under the ROC curve of a diagnostic model.** Area under the ROC curve of a diagnostic model based on MR radiomics features combined with clinical information and imaging features to construct a machine learning model in the training and test groups **Class 0** Benign group. **Class 1** HCC group. **Class 2** Malignant group
