# Supplementary material for: Research on multi-model imaging machine learning for distinguishing early hepatocellular carcinoma
Source: BMC Cancer. 2024 Mar 21;24:363. doi: 10.1186/s12885-024-12109-9 (PMC10956394; doi:10.1186/s12885-024-12109-9)
Supplement: Supplementary file 2 — Supplementary Material 2 [file 12885_2024_12109_MOESM2_ESM.docx]

**Table S1 CT radiomics features parameters**

| Plain phase | firstorder | Kurtosis |
| --- | --- | --- |
|  | firstorder | Minimum |
|  | firstorder | RootMeanSquared |
|  | glcm | Imcl |
|  | gldm | DependenceVariance |
|  | glrlm | LongRunHighGrayLevelEmphasis |
|  | glszm | LowGrayLevelZoneEmphasis |
|  | glszm | SmallArealowGrayLevelEmphasis |
|  | ngtdm | Busyness |
|  | ngtdm | Complexity |
|  | shape | Elongation |
|  | shape | LeastAxisLength |
|  | shape | Sphericity |
| Arterial phase | firstorder | RootMeanSquared |
|  | gldm | LargeDependenceEmphasis |
|  | glrlm | GrayLevelNonUniformityNormalized |
|  | glrlm | ShortRunLowGrayLevelEmphasis |
|  | glszm | SizeZoneNonUniformityNormalized |
|  | glszm | SmallAreaHighGrayLevelEmphasis |
| Portal phase | firstorder | Maximum |
|  | gldm | DependenceVariance |
|  | gldm | SmallDependenceLowGrayLevelEmphasis |
|  | glrlm | ShortRunHighGrayLevelEmphasis |
|  | glszm | GrayLevelNonUniformity |
|  | glszm | HighGrayLevelZoneEmphasis |
|  | glszm | LowGrayLevelZoneEmphasis |
|  | glszm | SizeZoneNonUniformityNormalized |
| Delayed phase | firstorder | Kurtosis |
|  | firstorder | Maximum |
|  | firstorder | Range |
|  | firstorder | Skewness |
|  | glcm | ClusterShade |
|  | glcm | Idmn |
|  | glcm | InverseVariance |
|  | gldm | LargeDependenceHighGrayLevelEmphasis |
|  | glrlm | RunVariance |
|  | glrlm | ShortRunHighGrayLevelEmphasis |
|  | glszm | GrayLevelNonUniformityNormalized |
|  | glszm | LowGrayLevelZoneEmphasis |
|  | glszm | SizeZoneNonUniformity |
|  | glszm | SmallAreaEmphasis |
|  | glszm | SmallAreaHighGrayLevelEmphasis |
|  | ngtdm | Contrast |

**Table S2 MR radiomics features parameters**

| Arterial phase | glszm | ZoneEntropy |
| --- | --- | --- |
| T2WI compression lipid | glcm | Correlation |
|  | glcm | InverseVariance |
|  | glcm | MCC |
|  | gldm | GrayLevelNonUniformityNormalized |
|  | gldm | LargeDependenceHighGrayLevelEmphasis |
|  | glszm | LowGrayLevelZoneEmphasis |
| DWI | firstorder | Minimum |
|  | glcm | Correlation |
|  | glcm | InverseVariance |
|  | glrlm | ShortRunEmphasis |
|  | glszm | ZonePercentage |
|  | shape | LeastAxisLength |
